# Case report: progressive familial intrahepatic cholestasis type 3 with compound heterozygous *ABCB4* variants diagnosed 15 years after liver transplantation

**DOI:** 10.1186/s12881-020-01173-0

**Published:** 2020-11-30

**Authors:** Mariam Goubran, Ayodeji Aderibigbe, Emmanuel Jacquemin, Catherine Guettier, Safwat Girgis, Vincent Bain, Andrew L. Mason

**Affiliations:** 1 Department of Medicine, University of Alberta Hospital, Edmonton, Canada; 2 Paediatric Hepatology & Paediatric Liver Transplant Department, Reference Center for Rare Paediatric Liver Diseases, FILFOIE, ERN RARE LIVER, Assistance Publique-Hôpitaux de Paris, Faculty of Medicine and University Paris-Saclay, CHU Bicêtre, Le Kremlin-Bicêtre, France; 3 Pathology Department, Assistance Publique-Hôpitaux de Paris, Faculty of Medicine and University Paris-Saclay, CHU Bicêtre, Le Kremlin-Bicêtre, France; 4 Department of Laboratory Medicine and Pathology, University of Alberta Hospital, Edmonton, Canada; 5 Division of Gastroenterology, 7-142 KGR, University of Alberta, Edmonton, Alberta T6G 2E1 Canada

**Keywords:** Progressive familial intrahepatic cholestasis, ABCB4, MDR3, Case report

## Abstract

**Background:**

Progressive familial intrahepatic cholestasis (PFIC) type 3 is an autosomal recessive disorder arising from mutations in the ATP-binding cassette subfamily B member 4 (*ABCB4*) gene. This gene encodes multidrug resistance protein-3 (MDR3) that acts as a hepatocanalicular floppase that transports phosphatidylcholine from the inner to the outer canalicular membrane. In the absence of phosphatidylcholine, the detergent activity of bile salts is amplified and this leads to cholangiopathy, bile duct loss and biliary cirrhosis. Patients usually present in infancy or childhood and often progress to end-stage liver disease before adulthood.

**Case presentation:**

We report a 32-year-old female who required cadaveric liver transplantation at the age of 17 for cryptogenic cirrhosis. When the patient developed chronic ductopenia in the allograft 15 years later, we hypothesized that the patient’s original disease was due to a deficiency of a biliary transport protein and the ductopenia could be explained by an autoimmune response to neoantigen that was not previously encountered by the immune system. We therefore performed genetic analyses and immunohistochemistry of the native liver, which led to a diagnosis of PFIC3. However, there was no evidence of humoral immune response to the MDR3 and therefore, we assumed that the ductopenia observed in the allograft was likely due to chronic rejection rather than autoimmune disease in the allograft.

**Conclusions:**

Teenage patients referred for liver transplantation with cryptogenic liver disease should undergo work up for PFIC3. An accurate diagnosis of PFIC 3 is key for optimal management, therapeutic intervention, and avoidance of complications before the onset of end-stage liver disease.

**Supplementary Information:**

The online version contains supplementary material available at 10.1186/s12881-020-01173-0.

## Background

Progressive Familial Intrahepatic Cholestasis (PFIC) syndromes represent a group of genetic disorders characterized by a defect in the secretion of bile components and bile acids [1]. PFIC disorders are numbered 1 through 5 based on the gene involved. They generally present in infancy or childhood with growth failure, coagulopathies due to impaired Vitamin K absorption, and progressive liver disease leading to cirrhosis before adulthood [2].

PFIC type 3 is an autosomal recessive disorder arising from mutations in the ATP-binding cassette subfamily B member 4 (*ABCB4*) gene which is located on chromosome 7 [3]. This gene encodes multidrug resistance protein 3 (MDR3), a phosphatidylcholine transporter (“floppase”) belonging to the family of adenosine-triphosphate-binding cassettes [4]. MDR3 localizes to the canalicular membrane of hepatocytes where it translocates phosphatidylcholine from the inner to the outer canalicular membrane [5]. Mutations in the *ABCB4* gene lead to impaired biliary phospholipid secretion. As a result, detergent bile salts are not inactivated by phospholipids. This causes injury to bile canaliculi and biliary epithelium that ultimately leads to cholestasis, cholangitis, ductopenia and biliary cirrhosis [6].

Variants in the *ABCB4* gene manifest with variable disease penetrance. For example, heterozygotes with functional MDR3 activity may simply manifest with increased serum gamma-glutamyl transferase (GGT), or present with a range of increasingly penetrant disorders, such as intrahepatic cholestasis of pregnancy, mild chronic cholangiopathy, or low-phospholipid-associated cholelithiasis [6]. Whereas a complete deficiency of MDR3 activity is more clearly linked with PFIC3 and decompensated biliary cirrhosis [6]. PFIC3 is the only PFIC associated with marked elevations in GGT [7]. The disease presents later in childhood or young adulthood and does not typically present with the intractable pruritus characteristic of both PFIC1 and PFIC2 [8].

Clinicians should suspect PFIC in infants and children presenting with cholestasis of unknown origin after the exclusion of more common causes such as Alagille syndrome, biliary atresia, sclerosing cholangitis, cystic fibrosis, and alpha-1 anti-trypsin deficiency [9]. Diagnosis of PFIC syndromes can be confirmed by genetic testing for all types and by immunostaining for PFIC 2 and 3. Ursodeoxycholic acid is the initial therapy of choice for children with all types of PFIC and is especially effective for PFIC3 patients with less severe disease [10]. However, liver transplantation is often the only alternative when pharmacological measures fail [11].

## Case presentation

### Patient presentation and timeline

We describe a 32-year old female with a retrospective diagnosis of PFIC type 3 that was made fifteen years after receiving liver transplantation for end-stage cryptogenic liver disease (see patient timeline).

The patient was first found to have hepato-splenomegaly at 18 months of age. The proband was otherwise healthy and had no family history of liver disease. No records could be found of investigations performed at the time. The patient then presented at age 14 with jaundice and fatigue and was found to have liver cirrhosis of unknown etiology. An abdominal ultrasound revealed evidence of cirrhosis, portal hypertension and incidental cholelithiasis. Liver biopsy showed micronodular cirrhosis with significant bile stasis in the hepatocytes. There was no significant iron staining, nor typical alpha-1 antitrypsin inclusions identified, but there was increased copper staining, consistent with cholestasis without other evidence of Wilson disease. A viral etiology was also excluded as the patient was confirmed to be immune to hepatitis B virus, and the hepatitis C serology was negative.

**Fig. 1:**
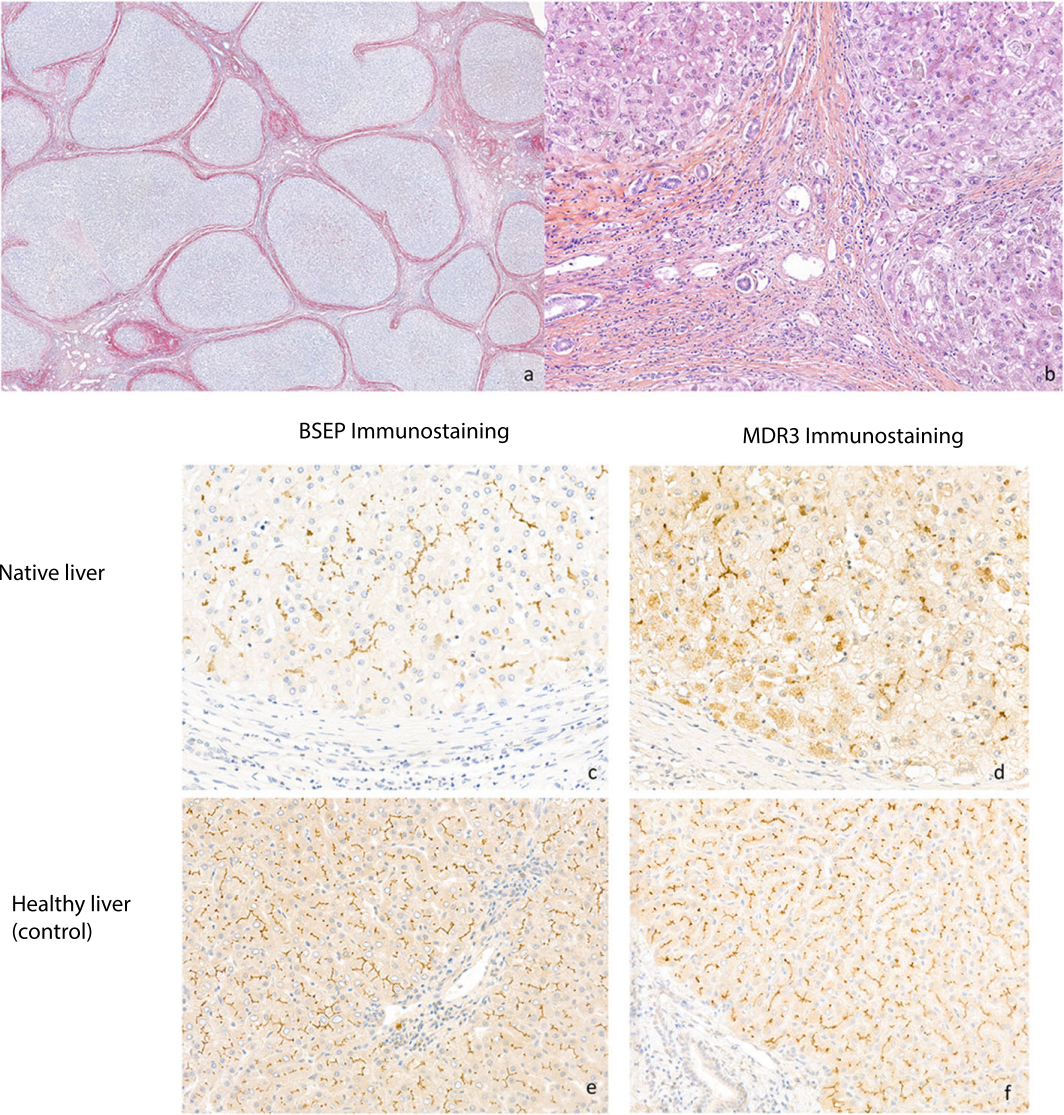
**a** Histology of explanted native liver showing biliary cirrhosis with annular fibrosis delimiting irregular parenchymal nodules (PicroSirius staining, × 15) and **b** moderate septal inflammation with some ductular reaction, canalicular bilirubinostasis and cholestasis changes in periseptal hepatocytes (H&E staining, × 120).**c** Immunochemistry study of the patient’s explanted native liver showing typical and even canalicular distribution of BSEP staining; **d** whereas the pattern of MDR3 canalicular staining is inconsistent and granular cytoplasmic signal observed within a proportion of hepatocytes. Within the control healthy liver, **e** regular canalicular BSEP and **f** MDR3 staining were observed

Over the following 3 years, a hepatic diagnosis was not found and the patient progressed to end-stage liver disease. The proband suffered from delayed growth and puberty as well as multiple complications of liver failure. These included jaundice, edema, ascites, two episodes of spontaneous bacterial peritonitis, splenomegaly, esophageal varices, coagulopathy, and hepatic encephalopathy. The patient was referred to the University of Alberta Liver Transplant program for a workup. At the time, her laboratory tests revealed an elevated serum AST of 174 (normal < 40 U/L), ALT of 64 (normal < 50 U/L), and total bilirubin levels of 491 (normal < 20 μmol/L), whereas serum GGT was not measured prior to transplantation. At age 17, the patient underwent a cadaveric liver transplantation and splenectomy for end stage liver disease. The histological evaluation of the native liver demonstrated biliary cirrhosis with moderate portal inflammation, ductular reaction, minimal loss of small bile ducts and chronic cholestasis. There was no biopsy evidence suggestive of sclerosing cholangitis such as a concentric “onion like” fibrosis nor fibro-obliterative scar of the bile ducts (Fig. 1a and b).

Post-operative course was complicated by obstructive jaundice due to a stricture within the common bile duct, which required stenting. One month after transplant, the patient experience elevated hepatic biochemistry tests and the liver biopsy was consistent with a mild acute cellular rejection, which was successfully treated with a steroid taper. The patient remained on immunosuppressive therapy post-transplant with tacrolimus to achieve levels of 10–12 μg/L (median tacrolimus level, 11.3 μg/L) as well as low dose mycophenolate. The latter was discontinued at 1 year following transplantation, due to bone marrow suppression.

Two years after liver transplantation, at age 19, the patient presented with a new thyroid nodule. A fine needle aspiration revealed papillary thyroid carcinoma, which was successfully treated with thyroidectomy and iodine ablation. The patient had no further complications from immunosuppression.

The proband’s clinical status and liver enzymes were stable until eleven years after liver transplant, at age 29, when routine follow-up labs revealed rising liver enzymes and bilirubin. The patient was asymptomatic at that time. Liver biopsy revealed pathological findings consistent with late cellular ductopenic rejection. Low-dose prednisone (20 mg daily) and azathioprine 100 mg daily were subsequently added to the tacrolimus (2.5 mg BID, median tacrolimus level of 9.1 μg/L). The patient complained of increasing fatigue and xerostomia. The liver enzymes continued to rise despite high dose immunosuppressive therapy and then improved following a trial of ursodeoxycholic acid (500 mg BID).

Fifteen years post-transplant, further biopsies revealed the patient has ongoing severe ductopenia (80%) with a component of plasma cell rich rejection. Serum markers for autoimmune hepatitis and primary biliary cholangitis were repeatedly negative.

At that point, the patient’s pretransplant hepatic diagnosis was still unknown. The initial histology had revealed biliary cirrhosis but no classical features or serum markers diagnostic of an immune mediated cholangiopathy such as primary sclerosing cholangitis or primary biliary cholangitis. We therefore hypothesized that the patient’s original disease was due to a congenital deficiency of a biliary transporter protein. This hypothesis raised the possibility that the ductopenia observed in the allograft could be due to an autoimmune response to the same biliary protein acting as a neoantigen not previously encountered by the immune system. This phenomenon has been demonstrated in some patients undergoing liver transplantation for PFIC, who subsequently develop alloimmune responses to canalicular proteins [12]. We therefore investigated whether the proband harbored a genetic defect to account for the biliary cirrhosis and sought evidence of alloimmune serum reactivity to the allograft.

### Genetic testing and immunohistochemistry

A DNA sample from the patient was sent to Eurofins Clinical Diagnostics for evaluated for variants within 76 genes within the Genetic Cholestasis panel [13]. The patient was found to have variants in the *ABCB4* gene  and both were described as variants of unknown significance (VOUS).

The first was c.3227G > A (p.S1076N) that was previously reported in neonatal cholestasis and intrahepatic cholestasis of pregnancy [14]. The second variant, c.3431 T > C (p.I1144T), has not been described to date.

To analyse the presence and position of these mutations, we employed nested PCR to clone and sequence product from both alleles. This was performed using outer primers (Forward: TCTCTCACCTTCATTTCACACC; Reverse: CTGAAGTATGGTGGTTTTGAGC) and inner primers (Forward: TTCAACTATCCCACCCGAGC; Reverse: TGAAAGGATGTATGTTGGCAGC). We confirmed that these mutations are found on alternate alleles by PCR amplification of the *ABCB4* region encompassing both variants. These data indicated that the patient was indeed a compound heterozygote.

We performed immunohistochemistry to determine the expression of the MDR3 protein and the bile salt export pump (BSEP) in the patient’s explanted native liver as compared to a healthy liver sample. BSEP is localized in the hepatic canaliculi and is encoded by the *ABCB11* gene, which is mutated in patients with PFIC2 [15]. A canalicular expression of BSEP was observed in the explanted patients’ native liver and this was comparable to the pattern observed in the healthy liver control (Fig. 1 c and e). In contrast, the MDR3 expression in the explanted native liver differed from the healthy liver control with a mixed signal of regular and dispersed canalicular pattern associated with a granular signal from within the hepatocytes (Fig. 1 d and f).

Given the mixed targeting of MDR3 to the canalicular membrane, the patient was probably not immunologically naïve to MDR3 protein. Nevertheless, we performed further studies using the patient’s serum to assess for alloimmunization. An immunofluorescence study was performed using the patient’s post liver transplant serum on normal human liver that revealed no reactivity to MDR3. While alloimmunization against MDR3 causing disease recurrence in allografts is theoretically possible, it has only been reported to occur in patients undergoing liver transplantation for PFIC2 associated with development of antibody reactivity to BSEP [12].

Taken together, the clinical presentation, the genetic testing, and the MDR3 distribution on both the canalicular membrane and within hepatocytes, were consistent with a diagnosis of PFIC3 disease. However, the patient has developed progressive ductopenic rejection with liver failure and is currently re-listed for a second liver transplantation. By clarifying the original hepatic diagnosis, we can conclude that the ductopenia in the allograft is due to chronic rejection rather than autoimmune in the allograft.

## Discussion and conclusions

Herein, we describe a patient with PFIC3 who ultimately required liver transplantation at the age of 17. This diagnosis was confirmed by retrospective analysis of the patient’s explanted native liver using immunostaining for MDR3 protein as well as genetic analysis of the *ABCB4* gene. It is probable that both mutations identified had a loss of function and contributed to the patient’s disease. One missense mutant (p.S1076N) likely leads to correct translocation of the protein to the canalicular membrane but with likely a loss of function. Indeed, it has been shown previously that the p.S1076C mutant identified is correctly targeted but has a significant loss of function [16]. The functional activity of the other missense mutant has not been previously described but this variant likely encodes a loss of function protein. The granular intracellular immunostaining suggests that the mutant protein is probably retained within the endoplasmic reticulum, as previously shown for a proportion of other MDR3 missense mutants [17].

Both mutations were previously described as variants of unknown significance which now may be of interest for biochemical study as targets for future therapies [16–18]. It has been suggested that gene therapy may become a potentially curative approach for affected individuals, as we gain a better understanding of the genotype-phenotype correlation in PFIC syndromes. Recently, two teams have successfully demonstrated stable ABCB4 gene expression and correction of PFIC3 phenotype in a murine model using adeno-associated virus serotype 8 (AAV8)-mediated gene therapy [19, 20]. Other studies have shown that molecular chaperones may be of therapeutic value in PFIC syndromes, including PFIC3 [21–25].

In retrospect, the clinical picture, laboratory results, and pathology data were al consistent with PFIC disease. The copper staining identified on the patient’s liver biopsies 3 years before transplant is a common finding in patients with cholestasis and PFIC 3 as a result of prolonged cholestasis [26, 27]. The PFIC3 diagnosis was not made prior to the patient’s presentation with end-stage liver failure and as such the patient did not receive ursodeoxycholic acid prior to transplantation which may have positively influenced the disease course. Ursodeoxycholic acid is the therapy of choice for children with PFIC and is especially effective for PFIC 3 with less penetrant disease [6]. Accordingly, this case report highlights the potential impact of exploring a diagnosis of PFIC 3 in teenagers referred for liver transplantation with cryptogenic cirrhosis with MDR3 immunostaining and *ABCB4* genetic analysis. An early diagnosis is critical for optimal management, therapeutic intervention, and avoidance of complications before the onset of end-stage liver disease.

## Supplementary Information

**Additional file 1.** Patient timeline

**Additional file 2.** CARE Checklist of information to include when writing a case report

## Data Availability

The datasets used and/or analysed during the current study are available from the corresponding author on reasonable request.

## Abbreviations

PFIC: Progressive familial intrahepatic cholestasis
MDR3: Multidrug resistance protein 3
GGT: Gamma-glutamyl transferase
VOUS: Variant of unknown significance
BSEP: Bile salt export pump
