# Supplementary material for: Case report: progressive familial intrahepatic cholestasis type 3 with compound heterozygous ABCB4 variants diagnosed 15 years after liver transplantation
Source: BMC Med Genet. 2020 Nov 30;21:238. doi: 10.1186/s12881-020-01173-0 (PMC7708126; doi:10.1186/s12881-020-01173-0)
Supplement: Supplementary file 1 — Additional file 1. Patient timeline [file 12881_2020_1173_MOESM1_ESM.docx]

| **Patient age** | **Event** |
| --- | --- |
| 18 months | Patient was found to have hepatosplenomegaly. |
| 14 years | Diagnosis of cryptogenic cirrhosis. |
| 14-17 years | Complications of cirrhosis and end stage liver disease. |
| 17 years | Cadaveric liver transplantation |
| 19 years | Diagnosis of papillary thyroid carcinoma, treated with ablation and thyroidectomy. |
| 28 years | Chronic rejection, patient started on UDCA |
| 32 years | Diagnosis of PFIC3 is made.  Patient is being re-listed for a second liver transplantation due to ongoing ductopenic rejection. |

**Case report: progressive familial intrahepatic cholestasis type 3 with compound heterozygous *ABCB4* variants diagnosed 15 years after liver transplantation**

**Patient timeline**
