# Supplementary material for: Case report: progressive familial intrahepatic cholestasis type 3 with compound heterozygous ABCB4 variants diagnosed 15 years after liver transplantation
Source: BMC Med Genet. 2020 Nov 30;21:238. doi: 10.1186/s12881-020-01173-0 (PMC7708126; doi:10.1186/s12881-020-01173-0)
Supplement: Supplementary file 2 — Additional file 2. CARE Checklist of information to include when writing a case report [file 12881_2020_1173_MOESM2_ESM.pdf]

CARE case report guidelines

CARE Checklist of information to include when writing a case report

(cc) BY-NC-ND

Topic	Item	Checklist item description	Reported on Line
Title	1	The diagnosis or intervention of primary focus followed by the words "case report"	Line 1-2
Key Words	2	2 to 5 key words that identify diagnoses or interventions in this case report, including "case report"	Line 50
Abstract (no references)	3a	Introduction: What is unique about this case and what does it add to the scientific literature?	Line 47
	3b	Main symptoms and/or important clinical findings	Line 36
	3c	The main diagnoses, therapeutic interventions, and outcomes	Line 40
	3d	Conclusion—What is the main "take-away" lesson(s) from this case?	Line 47
Introduction	4	One or two paragraphs summarizing why this case is unique (**may include references**)	Line 45
Patient Information	5a	De-identified patient specific information.	Line 84
	5b	Primary concerns and symptoms of the patient.	Line 88
	5c	Medical, family, and psycho-social history including relevant genetic information	Line 86
	5d	Relevant past interventions with outcomes	N/A
Clinical Findings	6	Describe significant physical examination (PE) and important clinical findings.	Line 97
Timeline	7	Historical and current information from this episode of care organized as a timeline	Line 95
Diagnostic Assessment	8a	Diagnostic testing (such as PE, laboratory testing, imaging, surveys).	Line 138
	8b	Diagnostic challenges (such as access to testing, financial, or cultural)	N/A
	8c	Diagnosis (including other diagnoses considered)	Line 165
	8d	Prognosis (such as staging in oncology) where applicable	Line 167
Therapeutic Intervention	9a	Types of therapeutic intervention (such as pharmacologic, surgical, preventive, self-care)	Line 102, 123
	9b	Administration of therapeutic intervention (such as dosage, strength, duration)	Line 110
	9c	Changes in therapeutic intervention (with rationale)	Line 122
Follow-up and Outcomes	10a	Clinician and patient-assessed outcomes (if available)	Line 168
	10b	Important follow-up diagnostic and other test results	N/A
	10c	Intervention adherence and tolerability (How was this assessed?)	Line 102, 123
	10d	Adverse and unanticipated events	Line 114
Discussion	11a	A scientific discussion of the strengths AND limitations associated with this case report	Line 171
	11b	Discussion of the relevant medical literature **with references**.	Line 174
	11c	The scientific rationale for any conclusions (including assessment of possible causes)	Line 174
	11d	The primary "take-away" lessons of this case report (without references) in a one paragraph conclusion	Line 194
Patient Perspective	12	The patient should share their perspective in one to two paragraphs on the treatment(s) they received	N/A
Informed Consent	13	Did the patient give informed consent? Please provide if requested	Yes ☒ No ☐
